# Supplementary material for: Novel QTL for Low Seed Cadmium Accumulation in Soybean
Source: Plants (Basel). 2022 Apr 24;11(9):1146. doi: 10.3390/plants11091146 (PMC9102923; doi:10.3390/plants11091146)
Supplement: Supplementary file 1 [file plants-11-01146-s001.zip › Supplementary Table S1.pdf]

Novel QTL for Low Seed Cadmium Accumulation in Soybean

Nour Nissan[1,2], Julia Hooker[1,2], Arezo Pattang[1,2], Martin Charette[1], Malcolm Morrison[1], Kangfu Yu[3], Anfu Hou[4], Ashkan Golshani[2], Stephen J. Molnar[1], Elroy R. Cober[1], Bahram Samanfar[1,2]

[1] Agriculture and Agri-Food Canada, Ottawa Research and Development Centre, Ottawa, ON, Canada.
[2] Department of Biology and Ottawa Institute of Systems Biology, Carleton University, Ottawa, ON, Canada.
[3] Agriculture and Agri-Food Canada, Harrow Research and Development Centre, Harrow, ON, Canada.
[4] Agriculture and Agri-Food Canada, Morden Research and Development Centre, Morden, MB, Canada.

* Correspondence: bahram.samanfar@agr.gc.ca

Supplementary Table 1. Sequence of CAPS and dCAPS marker pairs used for the major Cd gene (*Cda1*), in addition to the restriction enzymes used for dCAPS digestion. The digested fragments correspond to either high or low Cd accumulators.

**CAPs**			
Names	Sequence	Enzyme	Fragment
**A SNP R**<br>**GmHMA3-4F**	AAGAGCTGAACCTGATATTt<br>CCTGCAGAAAAGGCAGTGAT	-	A band at about 147 bp indicates the presence of A SNP for High Cd [C]
**G SNP R**<br>**GmHMA3-4F**	AGAGCTGAACCTGATATTC<br>CCTGCAGAAAAGGCAGTGAT	-	A band at about 147 bp indicates the presence of G SNP for Low Cd [C]
**A SNP F**<br>**MC4R**	GACATCGGTATCTCAATGGa<br>AGTCAACACAGCAAGCCAGA	-	A band at about 220 bp indicates the presence of A SNP for High Cd [C]
**G SNP F**<br>**MC4R**	GACATCGGTATCTCAATGGG<br>AGTCAACACAGCAAGCCAGA	-	A band at about 220 bp indicates the presence of G SNP for Low Cd [C]
**dCAPs**			
**dCAP MC1 F**<br>**dCAP MC4R**	CCTGCATTAGCCACAGCTGACATCGGTATCTCAAT**C**G<br>AGTCAACACAGCAAGCCAGA	Taq1	2 bands indicate low Cd [C]
**dCAP MC2 F**<br>**dCAP MC4R**	CCTGCATTAGCCACAGCTGACATCGGTATCTCAAT**C**G<br>AGTCAACACAGCAAGCCAGA	HpyI88I	Low band is Low Cd and High band is High Cd [C]
**GmHMA3-4F**<br>**dCAP MC3 R**	CCTGCAGAAAAGGCAGTGAT<br>GCAAGAGCTGAACCTGA**G**ATT	Hinf1	Low band is Low Cd and High band is High Cd [C]
